# Chiropractic Spinal Adjustment Increases the Cortical Drive to the Lower Limb Muscle in Chronic Stroke Patients

**DOI:** 10.3389/fneur.2021.747261

**Published:** 2022-02-04

**Authors:** Muhammad Samran Navid, Imran Khan Niazi, Dina Lelic, Imran Amjad, Nitika Kumari, Muhammad Shafique, Kelly Holt, Usman Rashid, Asbjørn Mohr Drewes, Heidi Haavik

**Affiliations:** ^1^Mech-Sense, Department of Gastroenterology and Hepatology, Aalborg University Hospital, Aalborg, Denmark; ^2^Department of Clinical Medicine, Aalborg University, Aalborg, Denmark; ^3^Centre for Chiropractic Research, New Zealand College of Chiropractic, Auckland, New Zealand; ^4^Faculty of Health and Environmental Sciences, Health and Rehabilitation Research Institute, AUT University, Auckland, New Zealand; ^5^Department of Health Science and Technology, Centre for Sensory-Motor Interactions, Aalborg University, Aalborg, Denmark; ^6^Faculty of Rehabilitation and Allied Health Sciences, Riphah International University, Islamabad, Pakistan; ^7^Faculty of Engineering and Applied Sciences, Riphah International University, Islamabad, Pakistan

**Keywords:** chiropractic, stroke, transcranial magnetic stimulation, spinal adjustment, motor evoked potential

## Abstract

This study aimed to investigate the effects of a single session of chiropractic spinal adjustment on the cortical drive to the lower limb in chronic stroke patients. In a single-blinded, randomized controlled parallel design study, 29 individuals with chronic stroke and motor weakness in a lower limb were randomly divided to receive either chiropractic spinal adjustment or a passive movement control intervention. Before and immediately after the intervention, transcranial magnetic stimulation (TMS)-induced motor evoked potentials (MEPs) were recorded from the tibialis anterior (TA) muscle of the lower limb with the greatest degree of motor weakness. Differences in the averaged peak-peak MEP amplitude following interventions were calculated using a linear regression model. Chiropractic spinal adjustment elicited significantly larger MEP amplitude (pre = 0.24 ± 0.17 mV, post = 0.39 ± 0.23 mV, absolute difference = +0.15 mV, relative difference = +92%, *p* < 0.001) compared to the control intervention (pre = 0.15 ± 0.09 mV, post = 0.16 ± 0.09 mV). The results indicate that chiropractic spinal adjustment increases the corticomotor excitability of ankle dorsiflexor muscles in people with chronic stroke. Further research is required to investigate whether chiropractic spinal adjustment increases dorsiflexor muscle strength and walking function in people with stroke.

## Introduction

Stroke is the second-most common cause of death worldwide (1) and the leading cause of severe disability in adults (2). It has a high prevalence, affecting ~200 people per 100,000 (3) and often requires extensive rehabilitation, with high economic and social costs (4). Stroke-induced impairment in motor function is common, with almost half of stroke survivors having limitations in walking ability (5). Weakness in ankle dorsiflexor muscles, such as the tibialis anterior (TA) muscle, is one of the major causes of gait dysfunction in people with stroke (6, 7).

Multiple rehabilitation techniques, such as physical therapy, brain-computer interface-based approaches, and motor relearning techniques have been shown to enhance motor recovery after a stroke (8–10). Recent research has suggested that chiropractic spinal adjustment could be another possible approach to improve post-stroke motor recovery (11–13). Chiropractic spinal adjustment involves the application of specific high-velocity, low amplitude (HVLA) adjustments to the site of spinal subluxations, i.e., a spinal segment that is not moving appropriately and is characterized by tight vertebral muscles and tenderness to touch (14–16). The site of the spinal subluxation is identified by utilizing a combination of pathophysiologic indicators of spinal dysfunction (17).

In the past two decades, research has demonstrated that chiropractic spinal adjustment has a neural plastic effect on the central nervous system (CNS). Multiple studies have found that (a single session of) chiropractic spinal adjustment modifies central processing, including somatosensory processing, sensorimotor integration, motor control, and pain, suggesting that the chiropractic intervention can rapidly affect neural and neuromuscular function in multiple ways (11–13, 15, 18–32). The spine is the biomechanical and neurological connection between the brain and limbs, and there is evidence that changes in afferent signals from the spine alter central neural processing (23), impacting the motor control of the limbs (11, 18, 21, 33–35). The articles mentioned above are discussed in detail in a recently invited review (15). In particular, strength increases have been documented to occur following chiropractic HVLA adjustments to dysfunctional spinal segments (15).

Multiple studies have explored the mechanism responsible for such changes in strength following HVLA chiropractic adjustments. The recruitment patterns of lower motor neurons has been evaluated using the transcranial magnetic stimulation (TMS) induced stimulus-response (SR) curves for both upper limb and lower limb muscles (21). The HVLA spinal adjustments resulted in significant increases in the maximum motor evoked potentials (MEPs). The plateau of the SR curve (MEPmax) for both the upper and lower limb muscle increased significantly, accompanied by a significant increase for all components of the movement-related cortical potential (MRCP), including the early bereitschaftpotential (EBP), late bereitschaftpotential (LBP) and also the peak negativity (PN). The change in MRCP noted after the spinal adjustment intervention indicates a change in motor preparatory activity occurring primarily within the supplementary motor area of the brain (21). The results of this study indicate that the changes in muscle force output following spinal adjustments are at least in part occurring at the cortical level, because it leads to significantly larger MEPmax for TMS induced input-output curves for both an upper and lower limb muscle, with significantly larger amplitudes of MRCP components following the spinal adjustments, while no changes were observed in the spinal excitability measures (21). To further characterize such change in neural excitability observed after HVLA adjustment, Haavik et al. (36) constructed peristimulus time histogram (PSTH) and peristimulus frequencygram (PSF) using single motor unit recordings. This study confirmed that spinal adjustments induced a consistent shortening of the TMS-induced cortical silent period (CSP), that had been documented in multiple previous studies, and demonstrated increase in the amplitude of individual I-waves, i.e., TMS-evoked descending corticospinal activity originating from indirect or trans-synaptic activation of the pyramidal tract or corticospinal tract upper motor neurons (UMN's). Another recent study, using both high density surface electromyography (HD sEMG) and intramuscular EMG, explored further how chiropractic adjustments could induce neural excitability and increase muscle strength (37). This study found a significant increase in strength of the tibialis anterior (TA) muscle. Further analysis found that the TA motor unit action potential conduction velocity increased without changes in motor unit discharge rate (37). This further supports the mechanisms that chiropractic adjustment-induced increase in strength is, in part, due to change in intracortical neural excitability that leads to increased recruitment of larger, higher threshold motor units. However, most of these studies have been conducted in relatively healthy populations. Therefore, it was unclear exactly why or how increases in strength occurred in chronic stroke survivors with persistent problems in muscle activation following chiropractic adjustments. Therefore, this current study explored changes in TMS induced MEPs in a chronic stroke population.

The changes in motor control at the cortical level and neural plasticity can be assessed by TMS. It has been used in studies exploring the effects of chiropractic spinal adjustment (21, 30, 36). In individuals with subclinical spinal pain (SCSP), the spinal adjustments have been shown to increase MEPs for both an upper and lower limb muscle (21). There was an increase in maximum MEP (the top of the stimulus-response curve) only for the upper limb muscles, while for the lower limb, there was a shift of the entire stimulus-response curve to the left (21), indicating a change in net excitability (38). This study also recorded MRCPs which are known to originate at the cortical level. As there was an increase in MRCPs following spinal adjustment, it was suggested that the changes after the adjustment occurred at least partially at the supra-spinal level (21). Other studies have made the same hypothesis utilizing cortical-based V-waves and spinal H-reflexes (11, 18, 28). Following spinal adjustment, these studies showed increased muscle strength, large V-wave increases, and decreased H-reflexes in individuals with SCSP (28), elite Taekwondo athletes (18), and stroke survivors (11), suggesting that spinal adjustment affects motor preparation and commands from the cortical regions to have more efficient control over force production. Altogether, these studies suggest that chiropractic spinal adjustment affects central cortical processing which thereby increases motor control efficacy.

The improvement in muscle control and strength may be necessary for a variety of clinical populations but essential for persons who had a stroke since motor functionality is often affected by stroke, and its improvement has been identified as one of the top 10 research priorities by stroke survivors, caregivers, and clinicians (39). Therefore, investigating whether chiropractic spinal adjustment can increase muscle strength and impact walking function in people with stroke can be important for future research. In line with this importance, recently, Holt et al. (40) has found clinically significant improvements in motor function following a combination of chiropractic spinal adjustment and physical therapy. It is also postulated that spinal adjustment might affect neural functionality (12, 13) in people with chronic stroke.

Since it has been shown that chiropractic spinal adjustment affects motor control and its associated cortical processing, we hypothesized that chiropractic adjustment would affect the TMS response in stroke patients. To date, the effect of chiropractic adjustment on MEP has been investigated in people with sub-clinical spinal pain (21). However, it is unknown if chiropractic spinal adjustment can alter MEP in people with stroke. Therefore, this study aimed to elucidate further the neural plastic effects of a single session of chiropractic spinal adjustment on corticospinal excitability following spinal adjustment using MEPs in stroke survivors.

## Methods

We used a double-blinded, randomized controlled, parallel design in the study. The experiments were conducted at the Railway General Hospital in Rawalpindi, Pakistan. The study was approved by the Riphah International University Research Ethics Committee, Pakistan (ref # Riphah/RCRS/REC/000118). The New Zealand College Chiropractic Research Committee also approved the study. The study was conducted in accordance with the Declaration of Helsinki.

### Participants

Twenty-nine stroke patients (22 males, 56.7 ± 11.01 years old) participated in this study. The participants were recruited from the outpatient facility of the rehabilitation department at Railway General Hospital, where they were present for conventional physical therapy. All participants gave their written informed consent to participate in the study. The participant details are shown in Table 1.

**Table 1:** Patients' characteristics.

**No**.	**Group**	**Age (years)**	**Gender**	**Type of Stroke**	**Affected Hemisphere**	**FM Score**	**Time since event (months)**
1	Control	49	M	Ischemia	Right	18	17
2	Control	56	M	Ischemia	Left	90	3
3	Control	63	M	Ischemia	Right	24	37
4	Control	49	M	Ischemia	Right	69	6
5	Control	82	M	Ischemia	Left	68	14
6	Control	48	M	Ischemia	Right	88	34
7	Control	48	F	Ischemia	Right	92	78
8	Control	58	F	Hemorrhage	Left	73	23
9	Control	62	M	Ischemia	Left	82	7
10	Control	59	F	Ischemia	Right	46	60
11	Control	60	M	Ischemia	Left	64	6
12	Control	60	M	Ischemia	Left	56	35
13	Control	65	M	Hemorrhage	Left	83	5
14	Control	67	M	Hemorrhage	Right	32	154
15	Control	63	M	Ischemia	Right	85	7
16	Control	53	F	Ischemia	Left	80	44
17	Intervention	47	M	Ischemia	Left	64	19
18	Intervention	48	M	Ischemia	Left	61	47
19	Intervention	34	M	Ischemia	Left	45	42
20	Intervention	36	M	Hemorrhage	Right	32	52
21	Intervention	72	F	Ischemia	Left	83	4
22	Intervention	35	F	Ischemia	Right	19	3
23	Intervention	62	M	Ischemia	Left	82	7
24	Intervention	63	M	Ischemia	Right	68	4
25	Intervention	70	F	Ischemia	Right	89	179
26	Intervention	52	M	Hemorrhage	Right	65	71
27	Intervention	59	M	Ischemia	Left	88	11
28	Intervention	64	M	Ischemia	Left	88	4
29	Intervention	61	M	Ischemia	Right	73	34

*FM, Fugl-Meyer Score*.

Participants were introduced to the lab environment before enrolling in the study. Participants were eligible to participate in the study if it had been at least 12 weeks since they had suffered from a stroke and they had some level of lower limb motor impairment. Motor performance was assessed by Fugl-Meyer (FM) motor assessment scale (41). Participants were ineligible to participate if they showed no evidence of spinal dysfunction (i.e., presence of vertebral subluxation indicators identified by a chiropractor), had absolute contraindications to chiropractic spinal adjustment (including spinal fracture, atlantoaxial instability, spinal infection, spinal tumor, or cauda equina syndrome), or previously had a significant adverse response to chiropractic care. Furthermore, they were ineligible to participate if they had contraindications to magnetic stimulation, such as a history of epilepsy, pregnancy, or metal implants in the body. They were also ineligible to participate if they had no MEPs in response to the TMS for the paretic TA at rest.

### Experimental Protocol

Participants were randomly divided into two groups, chiropractic (*n* = 13, age = 54.1 ± 13.1 years, FM score = 65.9 ± 22.2) and control (*n* = 16, age = 58.9 ± 8.8 years, FM score = 65.6 ± 24.0), using minimization tool (QMinim, Telethon Kids Institute, Australia) based on age and Fugl-Meyer Score after the eligibility assessment (42). The study was single-blinded; therefore All participants were blinded to group allocation. The data analyst was also blinded by using letters A and B instead of the actual group names in the data files.

Each session consisted of recording MEPs elicited by TMS before and immediately after the intervention. During each session, the participants were seated comfortably in a chair and were asked to keep their eyes open and stay relaxed.

### Interventions

The chiropractic spinal adjustment and control interventions were similar to those used in previous studies (11–13, 23, 27, 28, 43) that have investigated the neurophysiological effects of chiropractic spinal adjustment. These adjustments were HVLA thrusts to the spine that rapidly stretch the surrounding paraspinal tissues and, in particular, the deep small paraspinal muscles. This results in a “bombardment” of proprioceptive input to the CNS that elicits the changes in central neural excitability and motor control changes. The same chiropractor performed the experimental and control interventions. To test the effectiveness of participant blinding, at the end of the session, the participants were asked if they had perceived that they had undergone active treatment (“yes” or “no”).

#### Chiropractic Spinal Adjustment

The participants in this trial were checked and adjusted by a chiropractor using standard chiropractic techniques. The chiropractor performed manual HVLA spinal adjustment to the spine or pelvic joints identified as being subluxated (14). The sites selected for spinal adjustment were based on the clinical indicators of spinal and pelvic dysfunction (17), which were: tenderness to palpation of the relevant joints; manual palpation for the restricted intersegmental range of movement; palpable asymmetric intervertebral muscle tension, and any unusual or blocked joint play and end-feel of the joints. Chiropractors routinely use these biomechanical characteristics as clinical indicators for chiropractic spinal adjustment (17). Multiple levels of the spine were adjusted in each participant if required. The chiropractic spinal adjustment visit lasted ~15 min.

#### Control Adjustment

The control intervention acted as a physiological control for possible changes occurring due to the cutaneous, muscular, or vestibular input that would have occurred with the passive and active movements involved in preparing a patient for chiropractic spinal adjustment. The chiropractor performed the same assessment for spinal and pelvic dysfunction as the chiropractic adjustment group. However, instead of applying manual HVLA spinal adjustment, the chiropractor simulated the spinal adjustment session by providing passive and active movements to the participant's head, spine, and body, in line with what was performed in the actual chiropractic adjustment session. Thus, the only difference between the intervention and control is the application of the HVLA thrusts to the dysfunctional spinal segments. Moreover, the control intervention acts as a control for the time it takes actually to perform the HVLA adjustments, and it acts as a control for the touch and movement of the participant that occurs as the chiropractor moves a participant into an adjustment setup. During the adjustment setups for these control interventions, the chiropractor was careful not to thrust on the spine or take a vertebral segment to end-range tension. The duration of control adjustment was similar to that of chiropractic spinal adjustment.

### Electromyography

MEPs were recorded from the most affected lower limb using surface electromyography (EMG) electrodes (20 mm Blue Sensor Ag-AgCl, AMBU A/S, Ballerup, Denmark). Two electrodes were placed ~2 cm apart on the belly of the TA muscle, and a ground electrode was placed on the distal end of the tibia. The EMG signals from these electrodes were amplified by OT EMG USB (OT Bioelectronica, Turin, Italy) at 4,000 Hz with a gain of 1,000.

### Transcranial Magnetic Stimulation

MEPs were elicited using a single-pulse TMS generated using a Magstim 200 (Magstim Company, Dyfed, UK). A figure-of-eight double cone-coil, placed in a posterior-anterior current direction over the cortical motor area, was used to deliver the magnetic stimulus. Initially, the optimal stimulation site and resting threshold (RTh) were determined. The optimal stimulation site was the location where the largest MEPs were evoked compared to adjacent areas. The optimal stimulation site was marked on the tightly-fitted neoprene cap with 1 × 1 cm grid secured to the participant's head to ensure placement of the coil at the same location throughout the experimental session. The RTh was defined as the lowest stimulator output, which elicited 5 out of 10 MEPs with a minimum amplitude of 50 μV. Twelve stimuli, separated by 5–7 s, were delivered at 120% of the RTh before and after the interventions.

Using Signal Software version 4 (CED, UK), the peak-peak amplitudes were extracted from each MEP and saved in an Excel (Microsoft Corporation, USA) file. The average of the 12 MEPs was computed for each participant at each time point (pre, post) and exported to R version 4.0.2 (R Core Team, Vienna, Austria) for further analysis. Differences across the two intervention sessions were evaluated on the absolute and relative scales. Mean MEP amplitude values for individual participants were transformed to relative changes in MEP sizes using the following formula:


(1) $$\begin{array}{l} 100\times \frac{MEP_{abs}^{post}-MEP_{abs}^{pre}}{MEP_{abs}^{pre}} \end{array}$$

### Statistical Analysis

Separate statistical models evaluated MEP amplitudes in absolute and relative units. A linear regression model was set up for the absolute units, which included intervention (Chiropractic, Control) as a dichotomous variable and baseline MEP amplitude as a continuous variable. The inclusion of baseline scores as a linear covariate enabled the model to account for any baseline differences. As the data were not normally distributed for the relative units, a robust linear regression model was set up, which included intervention (Chiropractic, Control) as a dichotomous variable. This model also included MEP amplitudes in absolute units at baseline as a linear covariate to adjust baseline differences.

Intervention-wise mean MEP amplitudes (MEP_abs_ and MEP_%_) estimated with the models have been reported, along with standard errors, 95% confidence intervals, and relevant hypothesis tests with a significance level set at 0.05. No adjustments were applied for multiple comparisons as this reduces type-I errors at the cost of increased type-II errors (44).

## Results

All recruited participants were eligible to participate in the study. Hence, data from all of these were used for the analysis. On average, 5.25 ± 2.05 levels of the spine were adjusted in the intervention group.

Participant blinding was considered successful. Only one participant out of 13 felt that the intervention was not active (i.e., chiropractic) in the spinal adjustment group. In the control group, 14 out of 16 considered that the intervention they received was active, i.e., they perceived that they received chiropractic adjustments, but in reality, they received control intervention.

### Between-Group Differences

The differences between the two groups at the post-intervention time-point after removing the pre-intervention differences are given in Table 2. The chiropractic spinal adjustment resulted in a larger MEP size (by 0.15 mV, 92%, *p* < 0.001) compared to the control intervention.

**Table 2:** Between group MEP amplitude differences.

**Units**	**Difference ±SE, 95% CI [Lower, Upper]**	**H0: Difference = 0 *z*-value or *t*-value [df]**	***P*-value**
Absolute (mV)	0.15 ± 0.04, [0.08, 0.23]	3.42 [26]	**0.0002**
Relative (%)	92.2 ± 25.8, [41.5, 143]	3.568	**0.0004**

*Significant effects (p < 0.05) are in bold text*.

*The differences were computed from the statistical models with baseline covariate value set at 0 mV. SE, standard error; CI, confidence interval; H0, null hypothesis; df, degrees of freedom; mV, millivolts*.

### Within-Group Estimates

**Figure 1:**
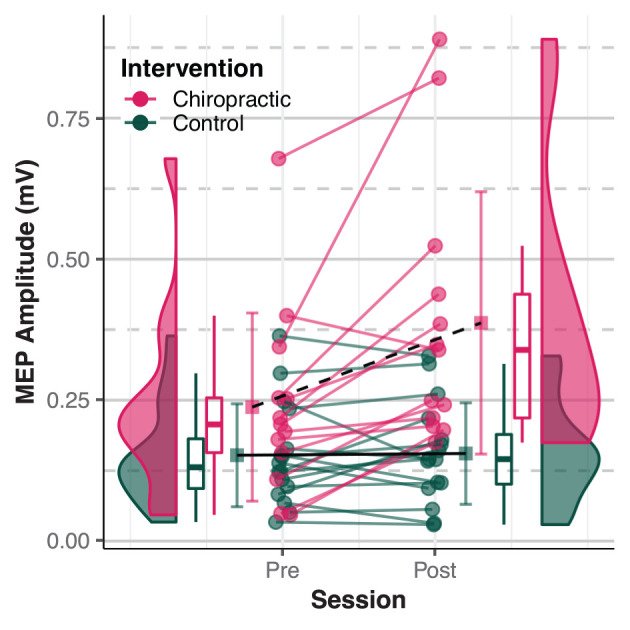
Motor Evoked Potentials (MEPs) amplitude. Dots represent individual MEP amplitudes. Boxplots show the median, 25th, and 75th percentiles. Error bars represent mean ± SD. The distribution plots show the density distribution estimated by a Gaussian kernel with an SD of 1.5. The chiropractic manipulation resulted in a larger MEP amplitude (dashed black line) compared to the control intervention (solid black line). The figure is inspired by raincloud plots (45).

**Figure 2:**
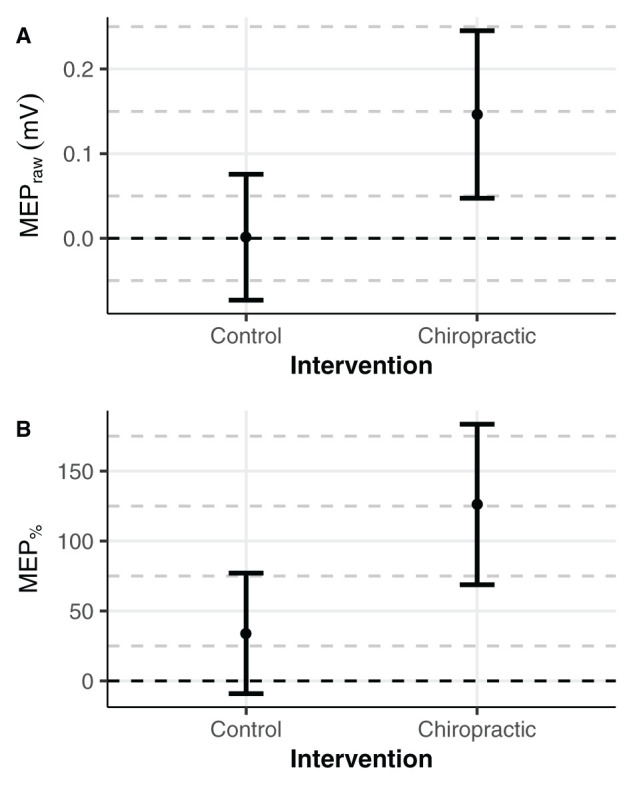
Estimated means of MEPs. Error bars represent mean ± 95 CI. The marginal means were estimated with pre-intervention values set to 0 (black dashed line). **(A)** Compared to post-control intervention, increased MEPs size was found after the chiropractic spinal manipulation by **(A)** 0.15 mV in absolute units and **(B)** 92% in relative units.

MEP sizes estimated from the statistical models in both absolute and relative units at the post-intervention time-point are given in Table 3. MEP amplitudes from individual participants are shown in Figure 1. The results suggested that the chiropractic adjustment elicited larger MEPs compared to the control intervention (Figure 2).

**Table 3:** Within-group MEP amplitude differences.

**Units**	**Intervention**	**MEP pre (mean ±SD)**	**MEP post (mean ±SD)**	**Mean ±SE, 95% CI [Lower, Upper]**	**H0: Mean = 0 *z*-value or *t*-value [df]**	***P*-value**
Absolute (mV)	Control	0.15 ± 0.09	0.16 ± 0.09	0.001 ± 0.04, [−0.07, 0.08]	0.03 [26]	0.974
	Chiropractic	0.24 ± 0.17	0.39 ± 0.23	0.15 ± 0.05, [0.05, 0.25]	3.04 [26]	**0.0054**
Relative (%)	Control	–	7.04 ± 43.8	34 ± 22, [−9.2, 77.1]	1.54	0.123
	Chiropractic	–	98.6 ± 99.8	126 ± 29.3, [68.7, 183]	4.303	**<0.0001**

*Significant effects (p < 0.05) are in bold text*.

## Discussion

### Main Findings

The primary outcome of this study was that chiropractic spinal adjustment increased the average MEP amplitude in both absolute (by 0.15 mV) and relative (by 126%) units. No differences in the MEP amplitude were seen after the control intervention. To the best of our knowledge, this study is the first to investigate and report changes in MEP following chiropractic spinal adjustment in people with stroke.

### Comparison With Previous Studies

The increased MEP amplitude following chiropractic spinal adjustment in this study implies increased excitability of motor pathways to the TA muscle. An increase in MEP size following chiropractic adjustment has been found in a recent crossover-designed study in people with SCSP (21). It was found that a single session of chiropractic adjustment significantly increased the MEP amplitude for the TA muscle and abductor pollicis brevis (APB) muscle by ~45 and 55%, respectively. The study further suggested that the changes after spinal adjustment were at the cortical level and could not be explained by changes at the spinal cord level. MRCPs, known to originate at the cortical level, were also increased following adjustments suggesting that the changes after the adjustment occur at least partially at the supra-spinal level (21). Changes in MEP's from lower limbs appear greater than upper limb MEP changes in several populations. Two crossover studies in people with SCSP found no difference in MEP amplitude recorded from the APB muscle following spinal adjustment at 150% active motor threshold (ATh) (ATh is defined as the minimal stimulus intensity at which 5 of 10 consecutive stimuli evoked an MEP with an amplitude of at least 100 μV while holding a weak isotonic background contraction of 5–10% of maximum voluntary contraction (MVC)) (30, 31). Thus, spinal adjustment increased the maximum MEP for the upper limb, whereas, for the lower limb, the entire stimulus-response curve increased (i.e., the curve shifted to the left) after spinal adjustment in an SCSP population (21). We, therefore, predicted that the stroke population would also see an increase in MEPs elicited at 120% of RTh, which is what was found in this study.

Changes in corticomotor excitability following chiropractic adjustment have also been reported by studies utilizing other TMS-induced outcome measures, such as the cortical silent period (CSP), short-interval intracortical inhibition (SICI), and short-interval intracortical facilitation (SICF). Two crossover studies in people with SCSP found a significant shortening of the CSP recorded from the APB muscle following a single session of chiropractic spinal adjustment (30, 31). The CSP used to be considered a cortical inhibitory phenomenon; however, using single motor unit data combined with surface EMG, Haavik et al. (36) found that chiropractic spinal adjustment increased the amplitude of I-waves during the shortening of the CSP in people with SCSP. A single TMS pulse evokes a series of descending corticospinal volleys that are separated from each other by about 1.5 ms [for review see (46)]. The evoked descending corticospinal activity has been directly recorded from epidural electrodes placed over the high cervical cord in animals and humans (46). The first wave is thought to be due to the direct activation of the axons or the axon hillock of fast-conducting pyramidal tract neurons (PTN) and is called the “D” wave (for direct activation) (47). The subsequent waves are thought to originate from indirect (i.e., trans-synaptic) activation of PTNs and are therefore termed “I” waves (48). These increased I wave amplitudes were shown to be actual excitatory events by constructing peristimulus frequencygram (PSF) (49, 50) from single motor unit recordings and noting that the discharge rates underlying these peaks were higher than the background (36). This, therefore, indicates that chiropractic adjustment can significantly increase the excitability of the motor pathways to low threshold motor units. Another study reported that a single session of chiropractic adjustment decreased SICI and increased SICF in the APB muscle and increased SICI and decreased SICF in the extensor indicis proprius (EIP) muscle (30). This study suggested a muscle-specific effect of chiropractic spinal adjustment on corticomotor excitability. Taken together, these findings suggest that chiropractic spinal adjustment leads to increased corticomotor excitability by modulating the balance of intracortical inhibitory and excitatory outputs to muscles, which can be the reason for the increased MEP amplitude found in this study and increased muscle strength and force production in previous studies (11, 18, 28, 37).

The evidence that chiropractic adjustment increases cortical drive is also supported by studies that have reported an increase in V-wave/Mmax ratio and V-wave amplitude for lower limb muscles following a single session of chiropractic spinal adjustment (11, 18, 28). The V-wave reflects supra-spinal input or cortical drive to the motor neuron pool (51, 52). Two crossover-designed studies reported an increase of 54 and 45%, respectively, in V-wave/Mmax ratio for the soleus muscle after a single session of chiropractic spinal adjustment in people with chronic stroke (11) and subclinical spinal pain (28). Another crossover-designed study in elite taekwondo athletes found that a single session of chiropractic adjustment significantly increased V-wave amplitude to the soleus muscle compared to a control intervention, and this change lasted for at least 60 min (18). Combined, the results of these studies and the current study show that chiropractic spinal adjustment can increase the cortical excitability of both plantar and dorsiflexor muscles. This increase in cortical excitability is probably the reason behind increased muscle strength following chiropractic spinal adjustment found in various populations such as Taekwondo athletes (18), people with SCSP (28, 37) and people with chronic stroke (11). Interestingly, in people with chronic stroke, the muscle strength increased by 65% after the adjustment (11).

Furthermore, chiropractic spinal adjustment-induced changes in all the above measures occurred with minimal or no change in the H-reflex (11, 18, 28) with no change in F-wave (21, 30). The H-reflex represents the excitability of the synapse between large, fast-conducting Ia fibres and lower motor neurons (53) and is mainly altered by presynaptic inhibition and lower motoneuron excitability (54). The F wave represents the antidromic activation of a portion of the lower motor neurons at the spinal cord level (53). This indicates that the changes seen are due to supra-spinal neuroplastic changes rather than changes in spinal excitability.

The increased MEP amplitudes found in this study can also indicate improved sensorimotor integration and functional connectivity in stroke survivors following spinal adjustment. The early peaks of somatosensory evoked potentials (SEPs) are severely decreased or even absent in stroke populations (55–59). However, Navid et al. (12) found increased N30 SEP peak amplitude in stroke survivors following spinal adjustment, implying improved early sensorimotor function. In addition, increased functional connectivity was found in the default mode network (DMN) in stroke survivors after a single session of spinal adjustment (13). Generally, decreased functional connectivity has been reported in the stroke population compared to healthy people (60–64). Therefore, an increased resting-state functional connectivity in the DMN is likely to be related to relieving of pain and improved memory (13) since decreased functional connectivity of the DMN is associated with chronic pain development and maladaptive neural plasticity (65, 66). Altogether, these cortical changes can also be the reason for increased MEPs measured at the TA muscle of the paretic limb.

### Possible Mechanisms

Motor recovery following a stroke depends on adaptive and maladaptive neural plasticity changes. As vertebral subluxations are central segmental motor control problems that cause ongoing maladaptive neural plastic changes in the CNS, correction of vertebral subluxation by chiropractic spinal adjustments results in central neural plastic changes (15, 23, 67, 68). Therefore, it is possible that the increase in MEP following chiropractic spinal adjustment observed in this study was due to changes in maladaptive neural plasticity.

The control intervention acted as a physiological control for possible changes occurring due to the cutaneous, muscular, or vestibular input that would have occurred with the passive and active movements involved in preparing a patient for chiropractic spinal adjustment. This was chosen to determine if the difference in outcome measure was due to the application of HVLA thrust or other cutaneous, muscular, or vestibular input caused by passive and active movements occurring during a chiropractic session. As is done before spinal adjustment, loading a joint has been shown to alter paraspinal proprioceptive firing in anaesthetized cats (69). Therefore, this was carefully avoided by stopping the movement before the end-range-of-motion when passively moving the participants, making the control adjustment appropriate.

### Study Considerations

There was no restriction on the type of stroke and the affected brain regions in the inclusion criteria. Therefore, there are possible differences in the brain morphology of the stroke patients and non-uniformity of the kind of stroke and affected brain regions. Thus, it is possible that we could have found even larger changes in MEPs if more homogenous patients with respect to stroke type and location were recruited. This would, of course, make the recruitment of individuals more difficult and limit the generalizability of the study. Furthermore, the onset of stroke was quite variable in the participants of the current study. Following a stroke, neuroplasticity is more pronounced in the first 3 months (70). Therefore, the effects of spinal adjustment may be different for different stages of stroke progression, which needs to be further investigated in future studies. Moreover, it is unknown if the observed changes in MEP depend on the severity or time since stroke and possibly contribute to the large variability observed in MEP amplitude. Therefore, future studies can explore this relationship and confirm the finding with other measures.

This was an exploratory study with a sample size of 29. However, previous studies based on stroke survivors have used sample sizes smaller than this study (10–21 participants) (11–13, 71–75). Future studies exploring the potential changes following chiropractic care for this population can consider increasing and diversifying the sample size to have more generalizable results.

This is a basic science study; therefore, the results from this study should not be extrapolated to clinical implications for the chronic stroke population. For clinical implication, Holt et al. (40) has found clinically significant improvements in motor function following a combination of chiropractic spinal adjustment and physical therapy. Further work is required to explore what potential clinical implications chiropractic care may have for different stages of stroke progression, which needs to be further investigated in future studies.

## Conclusion

The study results suggest that a single session of chiropractic spinal adjustment increases the corticomotor excitability in chronic stroke survivors by increasing the TA MEPs. Along with the previous findings, it can be postulated that such changes in corticospinal excitability improve muscle strength efficiency and function. Future studies should investigate the long-term effects of the adjustment in different stages and types of stroke for a better understanding of the impact of chiropractic care on stroke rehabilitation.

## Data Availability Statement

The numerical data supporting the conclusions of this article will be made available by the authors without, undue reservation. The Local Ethics Committee adhering to local data protection laws does not allow the sharing of patient's raw data.

## Ethics Statement

The studies involving human participants were reviewed and approved by Riphah International University Research Ethics Committee. The patients/participants provided their written informed consent to participate in this study.

## Author Contributions

MN, IN, DL, MS, AD, KH, and HH: conceptualization. MN, IN, IA, and MS: data curation. MN, UR, and IN: formal analysis and software. HH, DL, AD, and MS: funding acquisition. MN, IN, DL, MS, IA, and KH: investigation. MN, IN, DL, NK, MS, and AD: methodology. IN, DL, MS, AD, and HH: project administration. IN, MS, AD, and HH: resources. IN, DL, KH, AD, and HH: supervision. MN, IN, NK, UR, KH, and AD: validation. MN, UR, and NK: visualization. MN and NK: writing—original draft preparation. MN, IN, DL, NK, MS, KH, IA, UR, AD, and HH: writing—review and editing. All authors contributed to the article and approved the submitted version.

## Funding

This study was funded by the Centre for Chiropractic Research Supporters Program at the New Zealand College of Chiropractic.

## Conflict of Interest

The authors declare that the research was conducted in the absence of any commercial or financial relationships that could be construed as a potential conflict of interest.

## Publisher's Note

All claims expressed in this article are solely those of the authors and do not necessarily represent those of their affiliated organizations, or those of the publisher, the editors and the reviewers. Any product that may be evaluated in this article, or claim that may be made by its manufacturer, is not guaranteed or endorsed by the publisher.
